# Circulating PD‐1 (+) cells may participate in immune evasion in peripheral T‐cell lymphoma and chidamide enhance antitumor activity of PD‐1 (+) cells

**DOI:** 10.1002/cam4.2097

**Published:** 2019-04-10

**Authors:** Wei Zhang, Haorui Shen, Yan Zhang, Wei Wang, Shaoxuan Hu, Dongmei Zou, Daobin Zhou

**Affiliations:** ^1^ Department of Hematology Peking Union Medical College Hospital Beijing P.R. China

**Keywords:** adaptive immune, chidamide, innate immune, peripheral T‐cell lymphoma, programmed cell death‐1

## Abstract

Peripheral T‐cell lymphoma (PTCL) is a heterogeneous disease with poor outcomes. We intend to explore the role of circulating PD‐1 (+) cells in tumor immune evasion in PTCL patients and the mechanism of chidamide as a regulator of immune‐associated medicine on PD‐1 (+) cells. Gene expression profiling (GEP) was performed on circulating PD‐1 (+) cells from 22 PTCL patients and 13 healthy subjects, and circulating PD‐1 (−) cells from 2 PTCL patients. PD‐1 (+) cells were treated with chidamide, and the production IFN‐γ and cytotoxicity were analyzed. GEP were performed on circulating PD‐1 (+) cells from 2 PTCL patients treated with chidamide combined with chemotherapy and 1 patient treated with traditional chemotherapy. GEP showed that genes associated with innate immune response were abnormally expressed in PD‐1 (+) cells of PTCL patients compared with healthy subjects, meanwhile the expression of CTLA‐4 was significantly higher in PD‐1 (+) cells than that of PD‐1 (−) cells. In vitro study revealed decreased level of IFN‐γ secretion and impaired cytotoxic activity of PD‐1 (+) cells compared with PD‐1 (−) cells, while chidamide could recover the deficiencies and upregulate adaptive immune‐associated genes in PD‐1 (+) cells of PTCL patients. Our research indicated that PD‐1 (+) cells might have deficiencies in innate and adaptive immune response and chidamide may reverse the defects.

## 1 INTRODUCTION

Peripheral T‐cell lymphoma (PTCL) is a rare subentity of non‐Hodgkin's lymphoma (NHL), comprising only 5%‐10% of all lymphoid neoplasms.^1^ Nevertheless, this disorder is present at a higher prevalence in China (23%‐27%).^2^ Extranodal NK/T‐cell lymphoma, nasal type is the most commonly encountered PTCL subtype in China, followed by PTCL, not otherwise specified (PTCL‐NOS), anaplastic large cell lymphoma (ALCL) and angioimmunoblastic T‐cell lymphoma (AITL).^3^, ^4^ Chemotherapy remains the mainstay of treatment for PTCL, with frontline therapy based predominantly on anthracycline‐containing chemotherapy such as CHOP (cyclophosphamide, doxorubicin, vincristine, and prednisone) or CHOEP (CHOP plus etoposide), and is uniformly applied in all PTCL subtypes except Extranodal NK/T‐cell lymphoma, nasal type. However, there is an unacceptably high percentage (16%‐41%) of patients who experience progressive disease during induction or immediately after completing the course.^5^, ^6^

Tumor immunosurveillance system is a key mediator of antitumor effect by inhibition of carcinogenesis and maintenance of regular cellular homeostasis. Lymphocyte inhibitory receptors such as programmed cell death protein 1 (PD‐1) are crucial regulators of this delicate balance, and function to restrain tumor‐infiltrating lymphocytes (TILs) antitumor immunity.^7^ PD‐1 and its ligands are members of the CD28‐B7 family of receptor–ligand pairs. In tumor microenvironment (TME), the interaction between PD‐1 expressed on TILs and its ligands PD‐L1 on malignant tumor cells results in decreased cytokine output, including IFN‐γ and TNF‐α, leading to tumor evasion. These interactions suggest a dependence of tumor immune evasion on PD‐1 signaling and point to a therapeutic opportunity to restore antitumor immunity by PD‐1 blockade therapy.^8^

Significantly higher expression of PD‐1 on TILs in TME was observed in some patients with B‐cell NHL and related to disease stage and progression.^9^, ^10^ However, literature examining the relationship of PD‐1 in PTCL is scarce. One of the reasons for this is that it is difficult to obtain tumor tissue, to make matters more complex, PD‐1 has also been found to be expressed in TME tumor cells and is not exclusively found on TILs.^11^ Peripheral blood is an important and relatively stable environment for human immunity, and several studies have shown that treatment response and disease prognosis of patient with lymphoma is in part dependent on PD‐1 expression on peripheral blood lymphocytes.^12^, ^13^ Our previous study also found that PD‐1 was upregulated on lymphocytes of diffuse large B‐cell lymphoma (DLBCL) and PTCL patients, and the overexpression was related to disease stage and prognosis,^14^, ^15^, ^16^ indicating that PD‐1 (+) cells is a vital mediator in PTCL and may become a potential target for treatment.

Although PD‐1 inhibitor has demonstrated clinical efficacy in many malignancies, 40%‐60% of patients are refractory to this immune checkpoint inhibitor, and the mechanism is poorly understood. Several researchers found that histone deacetylase (HDAC) inhibitors can augment the response to immunotherapy with PD‐1 inhibitor.^17^, ^18^ Based on protein homology, HDACs have traditionally been described as belonging to one of the 4 classes: I, IIa, IIb, and IV,^19^ and play important roles in oncogenesis. Chidamide is benzamide class novel HDAC inhibitor and is approved by China food and drug administration (CFDA) for treating those suffering from refractory/relapsed PTCL.^20^ Our previous results showed that PD‐1 expression on peripheral blood lymphocytes was reduced in patients who responded to treatment of chidamide combined with chemotherapy,^16^ indicating that chidamide may exert its anti‐tumor potential by regulating PD‐1 (+) cell function.

This study seeks to characterize the genetic expression profiles and functions of peripheral blood PD‐1 (+) cells and how chiamide impacts circulating PD‐1 (+) cells of patients with PTCL in order to further explore the impact of circulating immune cells in tumor immunity.

## 2 MATERIALS AND METHODS

### 2.1 Patients

Peripheral blood was collected from patients who had been newly diagnosed with PTCL at PUMCH (Peking Union Medical College Hospital) between April 2017 and December 2017. All biopsies were described in compliance to the WHO (World Health Organization) classification^21^ and were analyzed by immunohistochemistry. Patients presenting with bone marrow involved, autoimmunity systematic disease or secondary cancer, infection of cytomegalovirus (CMV), or recurrent lymphoma following prior treatment were excluded. We performed flow cytometry on the peripheral blood of each patient to ensure that there were no tumor cells in the peripheral blood. The control group comprised of 13 healthy individuals that were recruited from the PUMCH Health Examination Center. All participants in this study provided signed and informed consent and ethical approval was obtained from the Ethics Committee of PUMCH.

First‐line therapy for patients with Extranodal NK/T‐cell lymphoma, nasal type is L‐asparaginase‐containing therapy such as SMILE (steroid, methotrexate, ifosfamide, L‐asparaginase, and etoposide) (N = 3) or GDP/ML (N = 2) (gemcitabine, dexamethasone, cisplatin, methotrexate, L‐asparaginase) regimen. Treatment with CHOP (cyclophosphamide, doxorubicin, vincristine, and prednisone) (N = 5) or CHOEP (CHOP with the addition of etoposide) (N = 8) regimen is the first line therapy for the other subtypes of PTCL patients. Four patients were treated with CHOP/CHOEP combined with chidamide.

### 2.2 The isolation of PD‐1 (+) and PD‐1 (−) cells

Peripheral blood mononuclear cells (PBMCs) were extracted from PTCL patients and healthy controls by Ficoll‐Hypaque density gradient centrifugation methods. About 10^7^~10^8^ mononuclear cells were harvested and subjected to a 20‐minute darkroom incubation period with PD‐1‐PE antibody at 4°C before being rinsed with 2 mLs of PBS and re‐incubated for another 20 minutes with anti‐PE microbeads at 4°C. Immunomagnetic beads were used to separate PD‐1 (+) and PD‐1 (−) cells. Ficoll was purchased from Haoyang, China (PKE122005X), anti‐PE microbeads (130‐048‐801) and PD‐1‐PE antibody (130‐117‐384, clone: PD1.3.1.3; PE) were purchased from MiltenyiBiotec, Germany.

### 2.3 Gene expression studies

Total RNA was extracted from PD‐1 (+) and PD‐1 (−) cells of patients with PTCL pusing TRIzol Reagent (Invitrogen, Carlsbad, CA, 15596018) in strict accordance to instructions supplied by the manufacturer's. NanoPhotometer® spectrophotometer (IMPLEN, CA) was used to verify the purity of RNA, and the Qubit® RNA Assay Kit in Qubit®2.0 Flurometer (Life Technologies, CA, Q32855) was used to quantify RNA concentration. Gene expression profiling (GEP) was conducted by Novogene company (Beijing, China). The amount of used RNA 200 ng. Read counts were adjusted by edgeR (3.12.1) program package through one scaling normalized factor prior to undergoing differential gene expression analysis of 2 conditions using the edge R R package (3.18.1). Benjamini & Hochberg methods were used to adjust *P* values, with an absolute foldchange of 2 and corrected *P*‐value of 0.05 determined as the criteria for a significantly differential expression. Average‐linkage hierarchical clustering was performed and heatmaps were generated. The cluster Profiler R package (3.0.5) was used to carry out GEP enrichment analysis. Gene Ontology (GO), Kyoto Encyclopedia of Genes and Genomes (KEGG) and Reactome pathway analysis was performed to classify genes into various categories and biological processes.

### 2.4 Cytokine measurement

PD‐1 (+) cells from 5 PTCL patients were seeded into 96‐well plates at 2 × 10^5^ cells per well. Chidamide was added to the cells at 2 different concentrations (0.3 and 0.5 μmol/L) 24 hours later. Cell supernatants were collected 48 hours later with Enzyme‐linked immunosorbent assay (ELISA) kits purchased from BD OptEIA™ (555142) were used to quantify IFN‐γ concentrations. Each individual experiment was repeated twice, with the results of each experiment obtained as a combination of three different experimental repeats.

### 2.5 Cellular cytotoxicity assay

Human K562 myeloid leukemia cells (National Infrastructure of Cell Resource, China, 3111C0001CCC000039) were used to evaluate cytotoxicity of PD‐1 (+) cells. PD‐1 (+) and PD‐1 (−) cells as effector cells were planted into 6‐well plates at 1.5 × 10^6^ cells per well and exposed to 0.3 μmol/L chidamide. After 48 hours, effector cells were washed with 1640 (Gibco, 11875093) and K562 cells were labelled with CFSE (ThermoFisher, C34554). Both target and effector cells were then co‐incubated in two different proportions (50:1 and 25:1 respectively) at 37°C; 4 hours later the test compounds were labeled with 7‐AAD (BD‐Pharmingen 559925) for 15 minutes at room temperature. Cellular cytotoxicity analysis was determined by FACScan flow cytometer (BD FACSCanto™, 6‐color). Percentage of killing rates was calculated as follows: CFSE (+) 7‐ AAD (+) cells/CFSE (+) cells ×100%. Each individual experiment was repeated twice, with the results of each experiment obtained as a combination of three different experimental repeats. CFSE and chidamide were dissolved in DMSO with the final concentration of DMSO being <1%.

### 2.6 Statistical analysis

SPSS 19.0 software and GraphPad Prism 7.0 (GraphPad Sofware, San Diego, CA) were used for statistical analysis and graphing. We performed a normal distribution test of Kolmogorov‐Smirnov and Q‐Q graphs for continuous variables, and the data conformed to normal distribution. Continuous variables were compared using Analysis of Variance (ANOVA), and categorical variables were compared using chi‐square test. Data are illustrated as mean ± standard deviation. Log‐transformation for ratio has been done and reanalyzed by ratio paired t test. Differences were considered statistically significant with a two‐sided *P* value of <0.05.

## 3 RESULTS

### 3.1 Patient characteristics

Twenty‐seven patients and 13 healthy controls were included in this study. Twenty‐two newly diagnosed PTCL cases were performed with GEP and 1 case was removed since the unqualified RNA concentration. Other 5 cases were tested for the function of PD‐1 (+) cells. The median age of 22 newly diagnosed PTCL patients (Table 1) was 44 years (18 to 71 years), and the male: female ratio of 1.75:1. Most patients were classified in clinical stage III—IV (63.6%). Based on the pathologic subtypes of lymphoma, Extranodal NK/T‐cell lymphoma accounted for majority (22.7%), followed by peripheral T‐cell lymphoma (non‐specific type) and Subcutaneous panniculitis like T‐cell lymphoma (SPTCL), which account for 18.2%, each. There were 13 individuals in the healthy control group and had a male: female ratio of 1.6:1 and a median age of 36 years (22 to 52 years).

**Table 1:** Baseline clinical characteristics of 22 PTCL patients

	Numbers (%)
Age	
≤60	19 (86.4)
＞60	3 (13.6)
Gender	
Male	14 (63.6)
Female	8 (36.4)
IPI	
＜2	10 (45.5)
≥2	12 (54.5)
Ann Anbor stage	
I~II	8 (36.4)
III~IV	14 (63.6)
Pathologic subtypes	
Extranodal NK/T‐cell lymphoma, nasal type	5 (22.7)
Peripheral T‐cell lymphoma, NOS	4 (18.2)
Subcutaneous panniculitis like T‐cell lymphoma	4 (18.2)
Anaplastic large‐cell lymphoma, ALK‐	2 (9.1)
Anaplastic large‐cell lymphoma, ALK+	2 (9.2)
Angioimmunoblastic T‐cell lymphoma	2 (9.3)
Enteropathy‐associated T‐cell lymphoma	2 (9.4)
Hepatosplenic T‐cell lymphoma	1 (4.5)

### 3.2 Differential gene expression between PTCL patients and healthy controls

**Figure 1:**
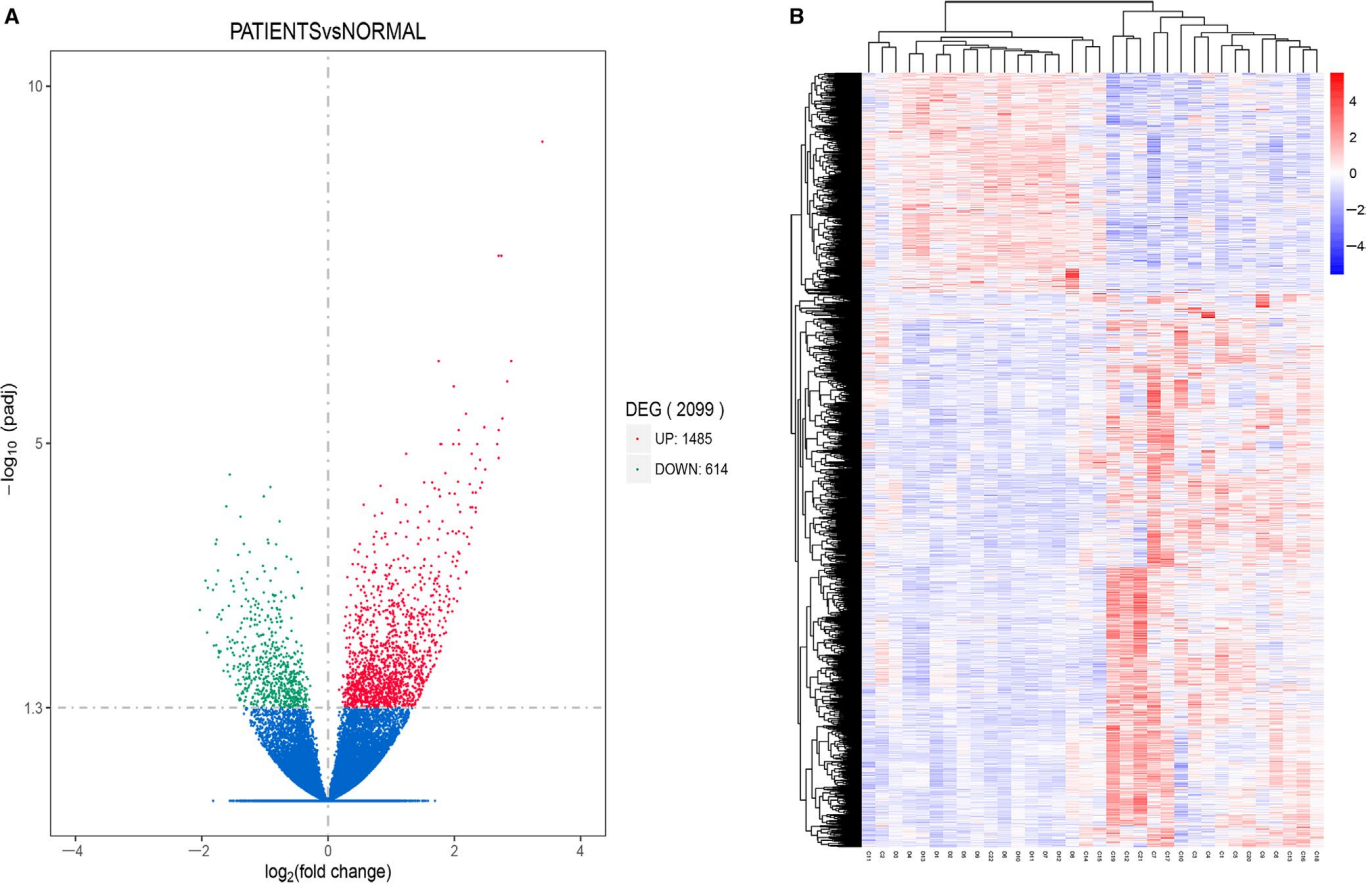
Differentially expressed genes between PTCL patients and healthy controls. A, Volcano Plot of gene expression. The abscissa indicates the log2(foldchange) value and the ordinate indicates padj. The green part shows the lower expressed genes and the red part shows the higher expressed genes in PTCL patients compared with the healthy controls. B, Hierarchical clustering heat map. The abscissa indicates the sample number, the ordinate indicates different gene probe. The rectangular units indicate the sample gene expression level, which are normalized by log10 (FPKM + 1), and red indicate high expression, while blue indicate low expression. The right side of the graph shows the color scale and the corresponding log10 (FPKM + 1) value

**Figure 2:**
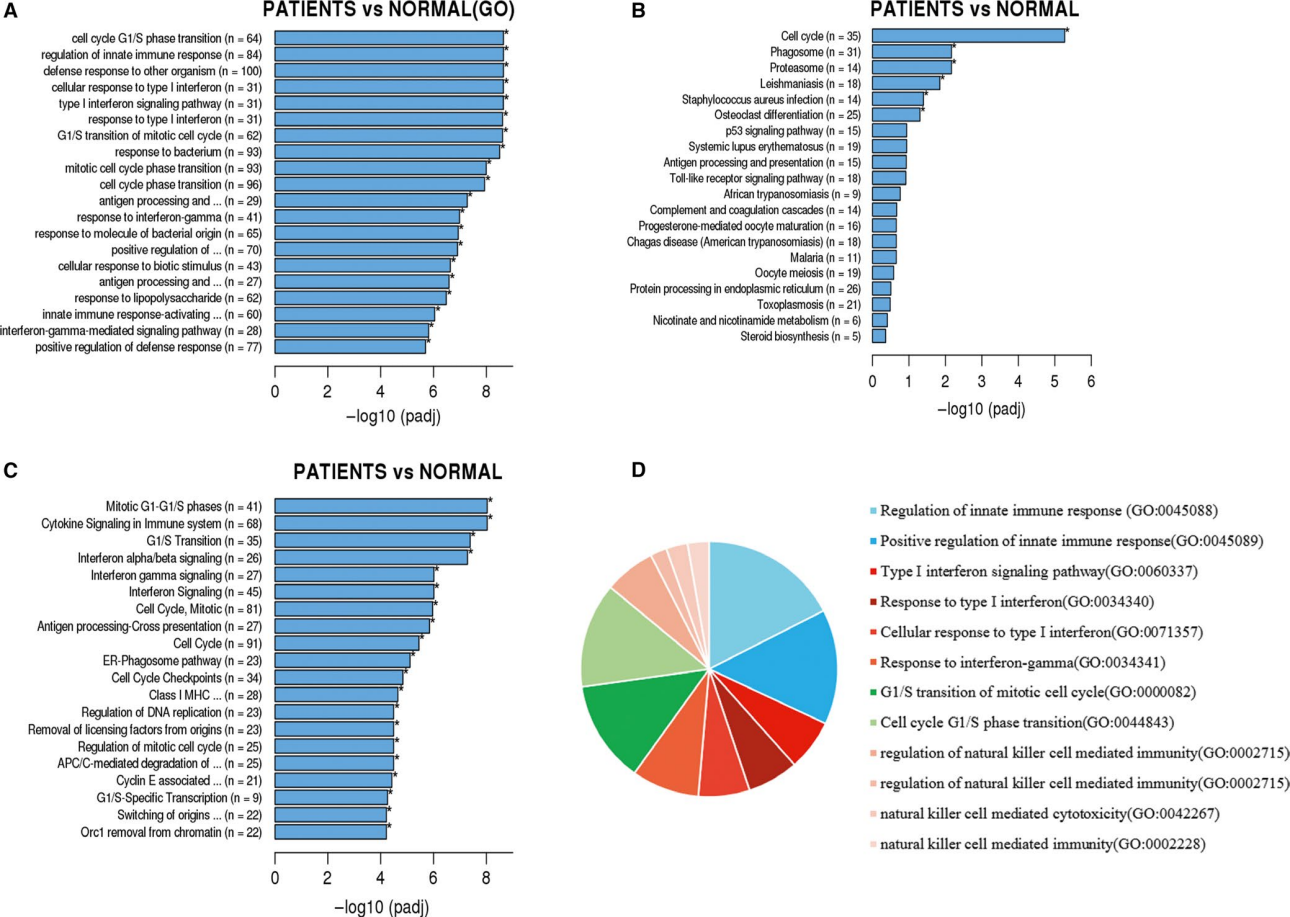
Enrichment analysis of differentially expressed genes by GO (A), KEGG (B), and Reactome (C). The pie chart shows (D) that the differentially expressed genes were mainly related to innate immune response, IFN‐γ pathways and cell cycle regulation

A heat map was used to illustrate the correlation coefficient between the healthy controls and different patient groups. There were 2099 differentially expressed genes in PD‐1 (+) cells in PTCL patients in comparison to healthy individuals, out of which 614 genes were found to be at a lower expression and 1485 genes were found to be highly expressed (Figure 1). These 2099 differentially expressed genes were further subjected to enrichment analysis using the GO, KEGG and Reactome package. Figure 2 demonstrates the significance of difference in part of the functional groups. Several of these functional groups were found to be involved in regulation of innate immune response (including phagosome processing, natural killer cell mediated cytotoxicity, etc), cell cycle regulation and IFN‐γ related pathways.

### 3.3 Gene expression and functional differences between PD‐1 (+) and PD‐1 (−) cells in patients with PTCL

GEP were used to explore the differential genes between PD‐1 (+) and PD‐1 (−) cells collected from 2 patients (C3 and C5) with PTCL. The results showed that genes associated with negative regulation of lymphocyte activation (GO:0051250) were expressed higher in PD‐1 (+) lymphocytes than PD‐1 (−) cells (including *CTLA4, TYRO3, SHH, TIGIT*), indicating that immune functions may be insufficient in PD‐1 (+) cells.

**Figure 3:**
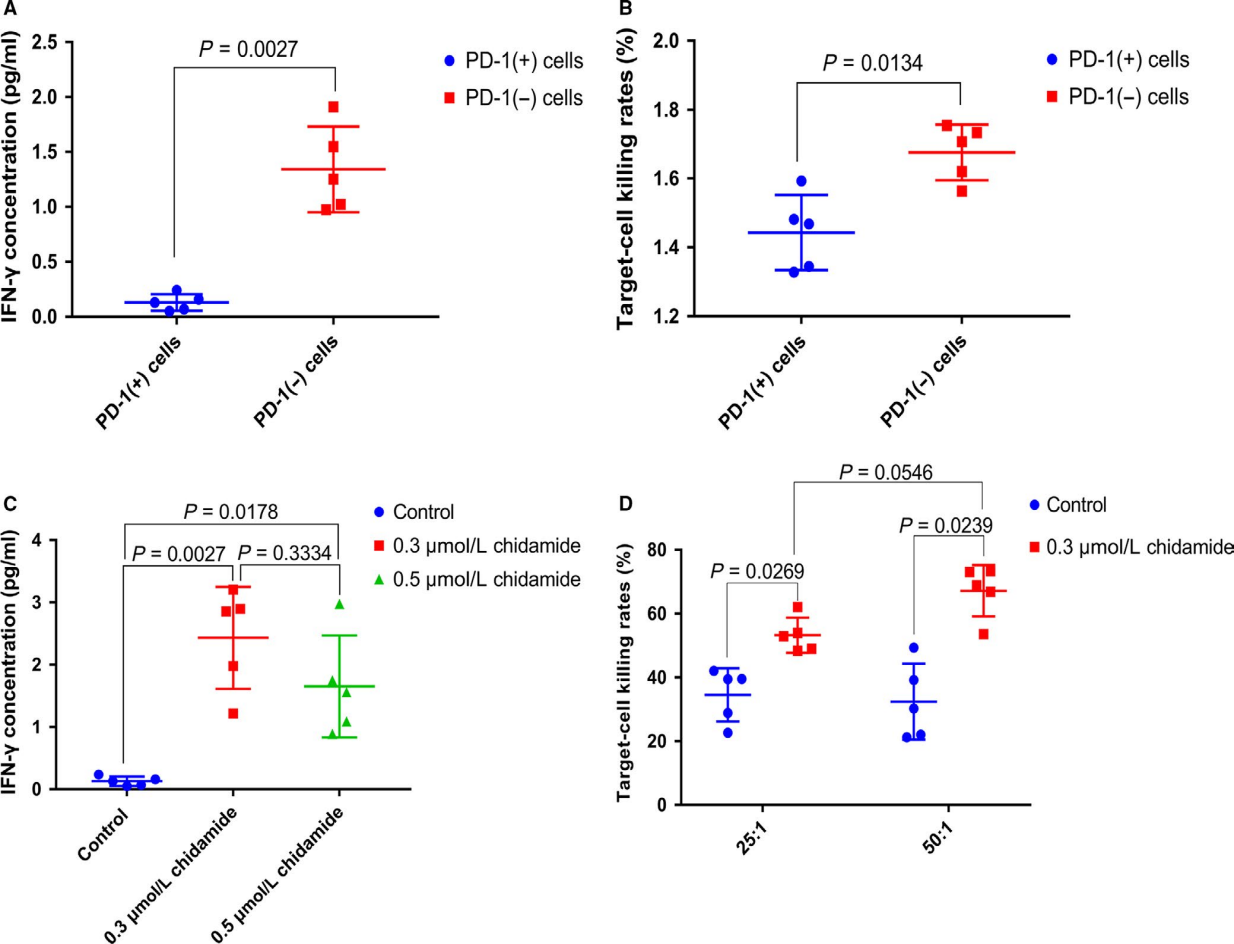
The capability of secretion of IFN‐γ (A) and the ability of injuring of K562 cells (B) of PD‐1 (+) cells was weaker than PD‐1 (−) cells. (C, D) demonstrated that chidamide could significantly enhance the ability of producing IFN‐γ and cellular cytotoxicity of PD‐1 (+) cells. a. concentration of IFN‐γ（pg/mL）: PD‐1 (+) cells 0.13 ± 0.03391, n = 5, PD‐1 (−) cells 0.9929 ± 0.1479, n = 5. b. target‐cell killing rates (%): PD‐1 (+) cells 32.46 ± 5.33, n = 5, PD‐1 (‐) cells 48.04 ± 3.837. n = 5. c. concentration of IFN‐γ (pg/mL): control 0.13 ± 0.03391, n = 5, 0.3 μmol/L chidamide 2.434 ± 0.3661, n = 5, 0.5 μmol/L chidamide 1.654 ± 0.3659, n = 5. d. targetcell killing rates (%): 25:1 group: control 34.56 ± 3.73, n = 5, 0.3 μmol/L chidamide 53.29 ± 2.459, n = 5,50:1 group: control 32.46 ± 5.33, n = 5, 0.3 μmol/L chidamide 67.17 ± 3.596, n = 5. The dots in the graphs represent biological repetitions

We then evaluated the immune system‐mediated antitumor effects of PD‐1 (+) and PD‐1 (−) cells derived from 5 patients with PTCL, the results showed that IFN‐γ was markedly raised in the supernatants of PD‐1 (−) cells when compared to PD‐1 (+) cells (0.9929 ± 0.1479 vs 0.13 ± 0.03391, *P* = 0.0027, (Figure 3A), meanwhile the cellular cytotoxicity of PD‐1 (−) cells was stronger than PD‐1 (+) cells (32.46 ± 5.33 vs 48.04 ± 3.837, *P* = 0.0134) (Figure 3B).

### 3.4 The influence of Chidamide on PD‐1 (+) cells

**Figure 4:**
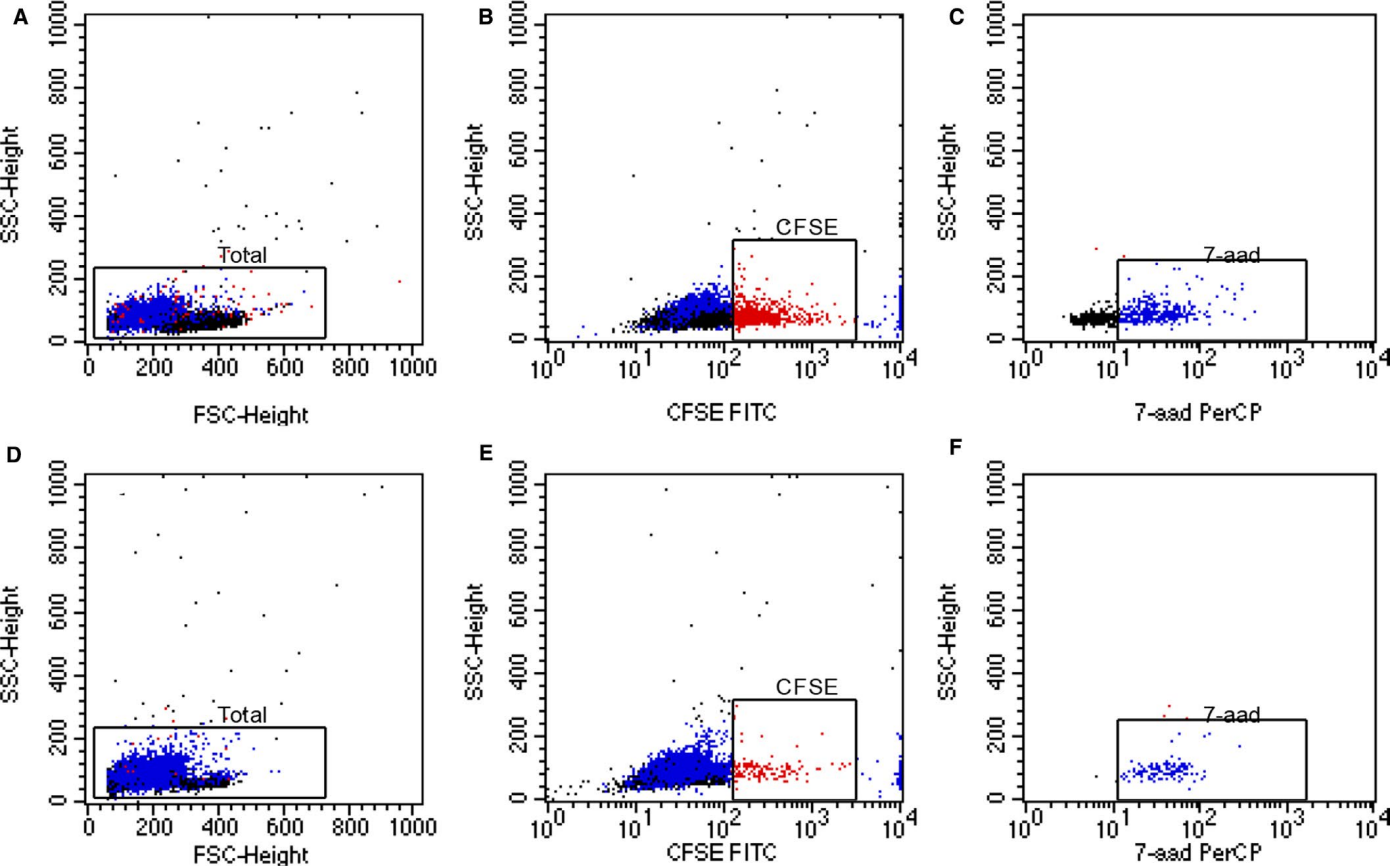
Flow cytometric measurement of cytotoxicity of PD‐1 (+) cells before (A‐C) and after treated with 0.3 μmol/L chidamide (D‐F) by the CFSE/7‐AAD assay. Effector cells and targeted cells gated on the basis of FSC‐H vs SSC‐H (A, C). CFSE was used to identify target cells (red plots in figure B and D) and 7‐aad was used to detect target cell death in the gated area (blue plots in figure C and F)

Chidamide was assessed for its effects on the immune system as a chemotherapeutic agent. IFN‐γ was significantly elevated in the supernatants of PD‐1 (+) cells treated with chidamide (Figure 3C). The PD‐1 (+) and PD‐1 (−) cells were incubated with 0.3 μmol/L chidamide for 48 hours and then co‐incubated with K562 human myeloid leukemia cells to further characterize cytotoxicity of PD‐1 (+) cells. Figure 4 demonstrated that chidamide could significantly enhance the ability of injuring of K562 cells of PD‐1 (+) cells. A target‐effector cell ratio of 50:1 was observed to confer the highest degree of target cell damage (Figure 3D).

Based on the above results, we found that chidamide could enhance the immune cell‐mediated anti‐tumor effect of PD‐1 (+) cells, so we further explored the changes of gene expression in PD‐1 (+) cells from PTCL patients prior and post treatment of chidamide. Of the 22 patients who underwent GEP analysis, a total of 11 patients completed treatment (C1, 3, 5, 6, 14, 16‐18, 19‐21) and C1, C3, C5, C6, C16, C18, and C20 had the similar gene expression patterns (Figure 1). C1, C3, C5, and C16 were treated with chidamide combined with traditional chemotherapy, only C1 died of disease progression, the other 3 patients all achieved at least PR C6, C18, and C20 received traditional chemotherapy, among them C20 achieved PR while the remaining patients underwent disease progression after treatment (Table 2).

**Table 2:** Clinical data of the 7 patients with PTCL

Number	Gender	Age (y)	IPI	AnnArbor	Pathologic subtypes	OS (months)	PFS (months)	Initial chemotherapy	Assessment of disease
C1	Female	61	5	IV B	Enteropathy‐associated T‐cell lymphoma	2	0	1 cycle of chidamide+CHOEP	PD
C3	Male	71	4	IV B	Anaplastic large‐cell lymphoma, ALK‐	9	9	8 cycles of chidamide+CHOP	PR
C5	Male	34	0	IE A	Subcutaneous panniculitis like T‐cell lymphoma	18	18	8 cycles of chidamide+CHOP	CR
C6	Male	53	3	IV B	Enteropathy‐associated T‐cell lymphoma	12	11	8 cycles of CHOEP	PD
C16	Male	27	3	IV B	Subcutaneous panniculitis like T‐cell lymphoma	12	12	8 cycles of chidamide+CHOEP	CR
C18	Male	55	1	II B	Extranodal NK/T cell lymphoma, nasal type	10	4	4 cycles of SMILE+radiation	PD
C20	Female	38	2	II B	Extranodal NK/T cell lymphoma, nasal type	15	15	6 cycles of GDP/ML+radiation	PR

CHOEP, CHOP plus etoposide; CHOP, cyclophosphamide, doxorubicin, vincristine, and prednisone; CR, complete remission.; GDP/ML, gemcitabine, dexamethasone, cisplatin, methotrexate, L‐asparaginase; PD, progressive disease; PR, partial remission; SMILE, steroid, methotrexate, ifosfamide, L‐asparaginase, and etoposide.

**Figure 5:**
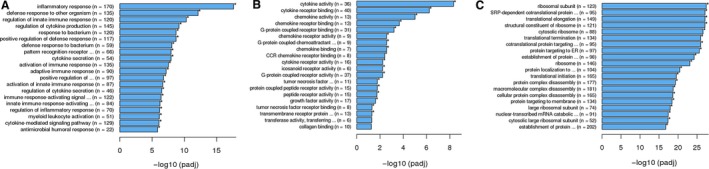
Enrichment analysis of variant genes of C3 (A), C5 (B), and C6 (C) patients before and after treatment

GEP was performed on peripheral PD‐1 (+) cells from C3,，C5, and C6 at diagnosis and the end of treatment or disease progression. As shown in Figure 5, genes related to inflammatory response (GO:0006954), regulation of innate immune response (GO:0045088), positive regulation of defense response (GO:0031349) were upregulated after treatment in C3, and genes associated with chemokine‐mediated signaling pathway (GO:0070098), cell chemotaxis (GO:0060326), cellular response to interleukin‐1 (GO:0071347) and leukocyte chemotaxis (GO:0030595) were upregulated after treatment in C5. The variations of genes in C6 after treatment were significantly different from C3 and C5. Genes involved in ubiquitin‐like protein‐specific protease activity (GO:0019783), ubiquitin hydrolase activity (GO:0036459), helicase activity (GO:0004386) were upregulated at the end of 8 cycles of CHOEP treatment (the disease progressed one month later).

## 4 DISCUSSION

Our results showed that genes associated with innate immune response and IFN‐γ pathway were abnormally expressed in PD‐1 (+) cells of PTCL patients compared with the healthy controls, meanwhile PD‐1 (+) cells demonstrated a markedly raised CTLA‐4 expression, an important inhibitory molecule for lymphocytes, in contrast to PD‐1 (−) cells in PTCL patients. Furthermore, in vitro study revealed that the level of IFN‐γ in supernatant and cytotoxic activity of PD‐1 (+) cells was lower than PD‐1 (−) cells. Taken together, these data indicated that the expression of genes related to both innate and adaptive immunity was compromised in circulating PD‐1 (+) cells of patients with PTCL, which may lead to insufficient anti‐tumor immune activity of the host immune system and contribute to tumor immune escape. These findings were in line with recent studies which have shown that immune abnormalities play an important role in tumor evasion, and provide rationale for the use of immune checkpoint inhibitors such as PD‐1 and CLTA‐4 inhibitors for the treatment of PTCL patients.^22^

Several studies have revealed that HDAC inhibitors can enhance anti‐tumor effect by regulating the expression of genes associated with innate immune response. Guerriero et al^22^ found that Class IIa HDAC inhibitor TMP195 can promote macrophages towards an anti‐tumor phenotype with enhanced capacity to activate cytotoxic T lymphocytes by regulating its gene expression in mouse model of breast cancer, combining TMP195 with PD‐1 inhibitor can significantly enhance the durability of tumor reduction. So we further investigated whether chidamide could modulate immune‐associated genes in PD‐1 (+) cells. Our results showed that 3 of 4 patients who were treated with chidamide combined with chemotherapy responded to the treatment (C3, C5, C16). Two of 3 patients in traditional chemotherapy group were in progressive disease (C6, C18). GEP analysis of C6 showed that the genetic changes were mainly related to ribosome‐related pathways, suggesting that C6 had abnormalities in gene transcription, protein translation and modification, while genes involved in immune response did not change significantly before and after treatment. In contrast, genes participated in innate and adaptive immune system pathway were upregulated in C3 and C5 after treatment with chemotherapy containing chidamide, suggesting that chidamide could normalize the patient's immune system, which is consistent with the results reported by Ning et al,^23^ who found that chidamide can stimulate human immune cell‐mediated tumor cell killing activity, which involves increased expression of genes and proteins for NK cell function. Although C20 was also treated with chidamide combined chemotherapy, this patient died of disease progressed rapidly within 2 months. One reason may be that the lesion was located in the small intestine, given that the risk of digestive tract hemorrhage was high of the patient, the chemotherapy doses were reduced. Meanwhile immune regulation is a long‐term process, compared with tumor‐targeted drugs such as CD20 monoclonal antibody; immunomodulators do not rapidly exert anti‐tumor effects. Altogether, our results indicated that traditional chemotherapy may not improve immune‐mediated anti‐tumor effects in patients with PTCL, while chidamide combined traditional chemotherapy can enhance immune‐mediated anti‐tumor effects of PD‐1 (+) cells. It also reflected that there may be synergistic effects between traditional chemotherapy and chidamide, chemotherapy may promote the release of tumor antigens and facilitate the immune regulation of chidamide.

In our research, the in vitro experiments showed that chidamide could enhance the function of PD‐1 (+) cells in direct killing of tumor cells, which may indirectly reflect the increasing activity of NK cells, and the increasing secretion of IFN‐γ also mirrored the activity of immune cells secreting anti‐tumor cytokines. Barry et al^24^ found that dendritic cells (DC)—NK interactions in tumor microenvironment are potentially prognostic in anti‐PD‐1 immunotherapy and that T‐cell antitumor responses are dependent on these innate immune cells. A recent study has shown that CD56, a marker associated with NK and NKT cells, is upregulated in peripheral blood of patients with metastatic melanoma who are responsive to anti‐PD‐1 immunotherapy.^25^ All these data suggest that NK cells play an important role in tumors, and our results also provided an evidence that NK cells with highly expressed PD‐1 may participate in tumor evasion of PTCL and chidamide could revitalize its antitumor activity. However, the cases in our study were limited; more researches were needed to confirm our results.

There are also shortcomings in our paper. The components of PD‐1 (+) cells are complex. We used flow cytometry to analysis the components of these cells, and the results showed the cells were composed of CD4+, CD8+ T cells, NK cells, monocytes and Treg cells. At present, we cannot confirm that each group of cells plays a specific role in the disease, but the human body as a whole, we can find the problem from a slightly macroscopic perspective. In the future work, we will perform flow cytometer to sort each group of cells, and perform single‐cell sequencing and functional tests. In addition, due to the small number of samples, we did not perform a separate analysis of PTCL subtypes, but it can be seen from the Pearson correlation coefficient map (Appendix Figure S1) that the gene expression patterns are similar in the patient group, C14, C18, C20, C21 patients are Extranodal NK/T‐cell lymphoma, nasal type, but C21 expression pattern is different from other patients, indicating that the expression pattern may not be related to the type of disease. In the future, we will expand the sample size, extend the follow‐up time, and compare the differences in gene expression between patients with different types and different prognosis.

In conclusion, our data showed that PD‐1 (+) cells were a group of immune‐deficient cells that played an important role in PTCL. The aberrant expression of genes related to innate and adaptive immune response and the undermined antitumor activity compared with PD‐1 (−) cells indicate that PD‐1 (+) cells might be involved in tumor immune evasion. More importantly, our findings reveal that chidamide could normalize tumor immunity by regulating immune‐associated genes. Altogether, these findings suggest that chidamide leveraged the immune‐mediated anti‐tumor effects of PD‐1 (+) cells, which could have potential synergistic effect with PD‐1 inhibitors or other immunotherapy. Future research should focus on exploring the optimal combinations between HDAC inhibitors and existing immunotherapeutic agents in order to maximize treatment benefit for PTCL patients.

## CONFLICT OF INTEREST

The authors report no conflict of interest.
