# Supplementary figures and images for: Circulating PD‐1 (+) cells may participate in immune evasion in peripheral T‐cell lymphoma and chidamide enhance antitumor activity of PD‐1 (+) cells
Source: Cancer Med. 2019 Apr 10;8(5):2104–13. doi: 10.1002/cam4.2097 (PMC6536954; doi:10.1002/cam4.2097)

# Pearson correlation between samples

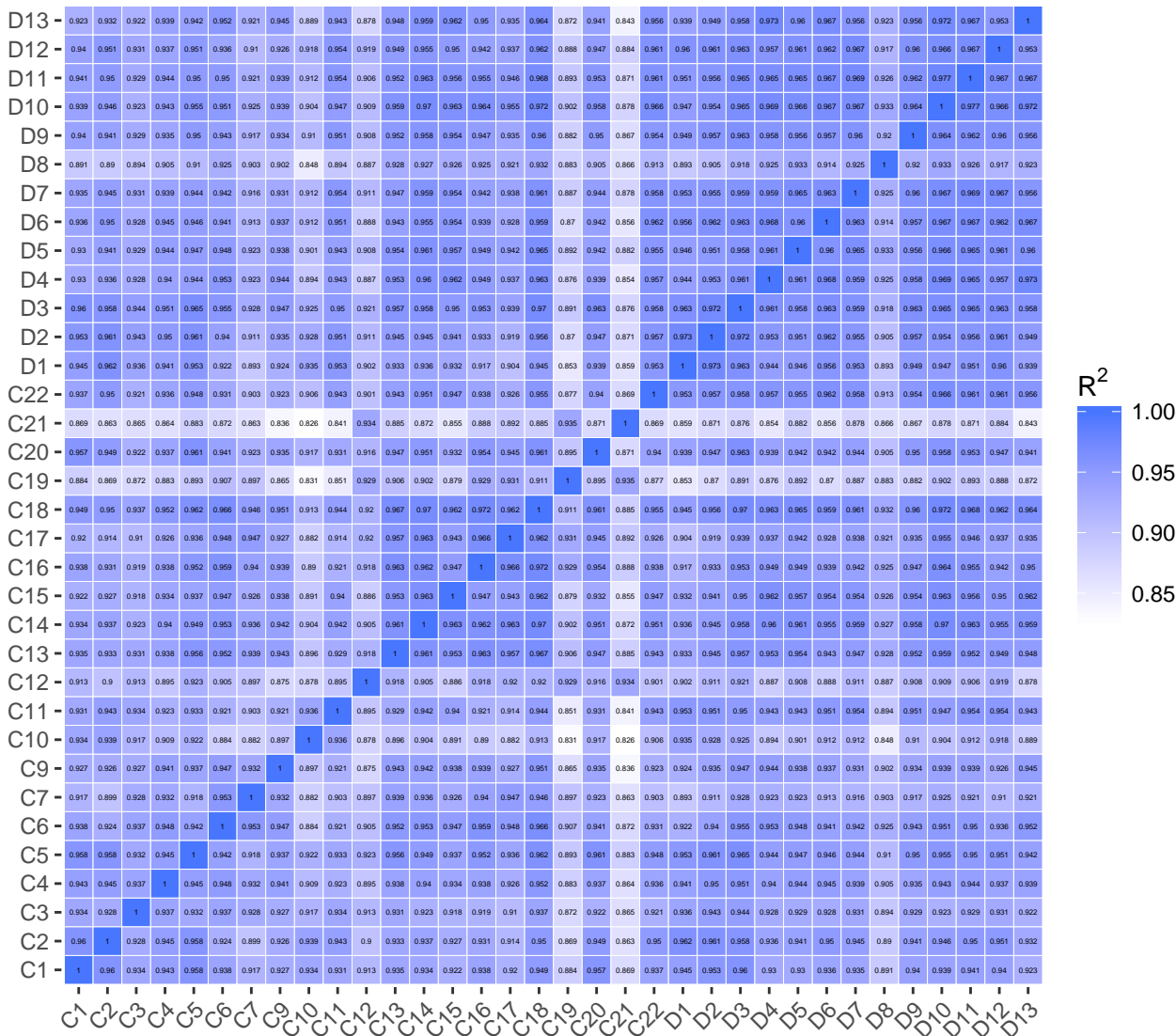

Supplement: Supplementary file 1 [file CAM4-8-2104-s001.pdf]
