# Supplementary material for: Circulating PD‐1 (+) cells may participate in immune evasion in peripheral T‐cell lymphoma and chidamide enhance antitumor activity of PD‐1 (+) cells
Source: Cancer Med. 2019 Apr 10;8(5):2104–13. doi: 10.1002/cam4.2097 (PMC6536954; doi:10.1002/cam4.2097)
Supplement: Supplementary file 2 [file CAM4-8-2104-s002.docx]

Appendix Figure 1. Pearson correlation between samples. Gene expression patterns are similar in the patient group, C14, C18, C20, C21 patients are Extranodal NK / T-cell lymphoma, nasal type, but C21 expression pattern is different from other patients.
